# The impact of vegetation phenology changes on the relationship between climate and net primary productivity in Yunnan, China, under global warming

**DOI:** 10.3389/fpls.2023.1248482

**Published:** 2023-09-18

**Authors:** Xu Chen, Yaping Zhang

**Affiliations:** ^1^Faculty of Geography, Yunnan Normal University, Kunming, Yunnan, China; ^2^The Engineering Research Center of Geographic Information System (GIS) Technology in Western China of Ministry of Education of China, Yunnan Normal University, Kunming, Yunnan, China; ^3^School of Information Science and Technology, Yunnan Normal University, Kunming, Yunnan, China

**Keywords:** net primary productivity, phenology, global warming, structural equation modeling, remote sensing

## Abstract

Climate influences net primary productivity (NPP) either directly or indirectly via phenology. Therefore, clarifying the indirect effects of climate on NPP through phenology is of utmost importance. However, the underlying mechanisms by which phenology indirectly affects NPP are unknown and poorly studied. Based on different structural equation models, this study analyzed the influence of phenology on the relationship between climate and NPP, and the results were as follows: (1) Temperature and solar radiation directly affect the end and beginning of the growing season, respectively, while precipitation indirectly affects the beginning of the growing season. (2) Spring phenology mainly affects the relationship between subsequent precipitation and net primary productivity, while autumn phenology mainly affects the relationship between temperature and net primary productivity. (3) Solar radiation is the most important direct influence factor on phenology and NPP, and the relationship between it and NPP is hardly disturbed by vegetation phenology. This research holds significant scientific and applied values in enhancing our understanding of the effects of global warming, forecasting ecosystem responses in the future, and formulating adaptation strategies.

## 1 Introduction

Net primary productivity (NPP) is defined as the amount of carbon fixed by plants through photosynthesis minus the amount used for respiration. It is a crucial indicator of plant community productivity and a major component of the terrestrial carbon cycle (Michaletz et al., 2014). NPP determines the net carbon input to terrestrial ecosystems, playing an important role in regulating global carbon (CO_2_) concentrations (Wang et al., 2021). Vegetation phenology refers to the recurring timing of plant life cycle events like germination, defoliation, flowering, and senescence. It signifies subtle changes throughout the vegetation growth cycle that are crucial for plant function, ecosystem services, and their biophysical and biogeochemical feedback to the climate system (Piao et al., 2019). Climate refers to the overall statistical characteristics of long-term atmospheric states and weather phenomena, such as precipitation, temperature, humidity, wind, and solar radiation. Studies have shown that climate is one of the main factors that directly impact vegetation phenology and NPP (Piao et al., 2019; Wu et al., 2021; Liu et al., 2022; Sun et al., 2022). Climate can strongly influence NPP by altering environmental conditions such as resource availability (nutrients, thermoregulation, water, and light) required for plant growth and photosynthesis; and also affecting the timing, duration, and magnitude of recurrent plant life cycle events (phenology), leading to changes in vegetation responses to seasonal fluctuations. For example, higher temperatures can stimulate plant metabolism and increase photosynthetic rates and NPP (Piao et al., 2019; He et al., 2022). Wang et al. (2023) found that NPP in Chinese forests showed a significant positive correlation with both mean annual temperature and mean annual precipitation, but this positive correlation gradually weakened over time. In addition, all plant phenological changes are almost highly correlated with temperature changes, especially in the months before seasonal life cycle events (Peñuelas and Filella, 2001). Warmer temperatures in spring can trigger earlier bud break, which extends the growing season and contributes to even greater plant productivity (Buermann et al., 2018; Zhou et al., 2020). Similarly, warmer temperatures in autumn may lead to delayed leaf senescence and extended periods of growth (Piao et al., 2019; Shen et al., 2022). However, higher temperatures increase respiration, which can wholly or partially offset productivity (Ruehr et al., 2023). On the other hand, lower temperatures can reduce photosynthetic rates, limiting plant growth. They can also delay critical events like bud break, ultimately shortening the available growing season for plants (Shen et al., 2022). And, this increased exposure to frost and cold temperatures may become a problem in colder regions (Piao et al., 2019). Furthermore, water limitation often decreases plant productivity, although not in all cases. Many ecosystems are becoming more sensitive to changes in water availability, in terms of productivity and greenness (Ruehr et al., 2023). Moreover, drought conditions and lower humidity can lead to plant water stress, causing earlier leaf senescence and a shorter growing season (Piao et al., 2019). For example, Ge et al. (2021) found that preseason droughts at the start of the vegetation growing season (SOS) are essential to vegetation productivity in the Yungui Plateau, southwest China.

Meanwhile, vegetation NPP is highly related to the timing of phenological onset, particularly in spring and autumn (Wu et al., 2021). The early SOS in spring and the delayed end of the vegetation growing season (EOS) in autumn, caused by climate warming, are major factors that increase plant productivity (Zhang et al., 2022). Seasonal changes in vegetation phenology can adjust photosynthesis and other ecosystem processes leading to changes in the timing and magnitude of NPP, which in turn has significant implications for terrestrial carbon cycling (Wu et al., 2021; Sun et al., 2022). For instance, the early SOS at the regional level can significantly boost terrestrial ecosystems’ carbon uptake in spring and potentially offset some of the increased atmospheric CO_2_ (Shen et al., 2022). Chen and Zhang (2023) found that SOS of vegetation phenology was negatively correlated with NPP in Yunnan, while EOS and LOS were positively correlated with NPP. Meanwhile, Sun et al. (2022) found a significant positive correlation between NPP and LOS in forests. With the increasing research on vegetation phenology and NPP, it has become evident that climatic factors can directly impact plant community NPP by regulating metabolic processes such as photosynthesis and respiration (Michaletz et al., 2014; Sun et al., 2022). Simultaneously, these factors also indirectly influence plant community NPP by governing phenological and growth status aspects, such as growing season length and plant size (Michaletz et al., 2014; Wu et al., 2021; Sun et al., 2022). Although Gao et al. (2023) found that in China, the direct effect of climate factors on NPP was stronger than the indirect effect, in some specific cases, these indirect effects can even contribute more to variation than direct effects (Zheng et al., 2020). For instance, in a study by Zhou et al. (2020), researchers found that spring vegetation growth across forests and grasslands was primarily determined by SOS rather than climatic factors. Additionally, Piao et al. (2019) observed that the extended duration of the vegetation growing season was the main factor contributing to enhanced productivity in boreal ecosystems since the 1980s. And Dang et al. (2023) also found that LOS was the most important factor driving vegetation productivity in the Northern Hemisphere ecosystems.

Finally, as mentioned above, climate change can indirectly affect the net primary productivity (NPP) of vegetation by influencing vegetation phenology changes (Wu et al., 2021). However, few studies have examined the relationship between climate change, phenology, and NPP (Sun et al., 2022). In particular, almost no studies have been conducted on climate affecting NPP indirectly through influencing phenology. Due to the diversity of vegetation phenology trends, the exact mechanism of how climate acts on phenology and thus indirectly affects NPP remains unclear (Yu et al., 2022). It remains challenging to properly assess the impact of climate change on vegetation phenology and NPP, and to quantify the relationship between vegetation phenology and NPP (Piao et al., 2019). Therefore, there is an urgent need to study how vegetation phenology affects the relationship between climate and NPP, i.e., the mechanism of how climate indirectly affects NPP by influencing phenology. This research finding can contribute significantly to our understanding of how phenology regulates NPP, thereby facilitating practices for carbon neutralization in China and globally (Sun et al., 2022; Yu et al., 2022). In this study, we aimed to investigate the temporal trends and variability of vegetation phenology in Yunnan under global warming conditions from 2001 to 2018, and to examine the mechanisms of NPP response to phenological changes under global warming. We aim to address the following key scientific questions: (1) What are the vegetation phenology patterns in Yunnan over the past two decades? (2) What are the mechanisms by which NPP responds to vegetation phenology changes? In particular, how does vegetation phenology impact the effects of climate change on NPP?

## 2 Data and methods

### 2.1 Research area

**Figure 1:**
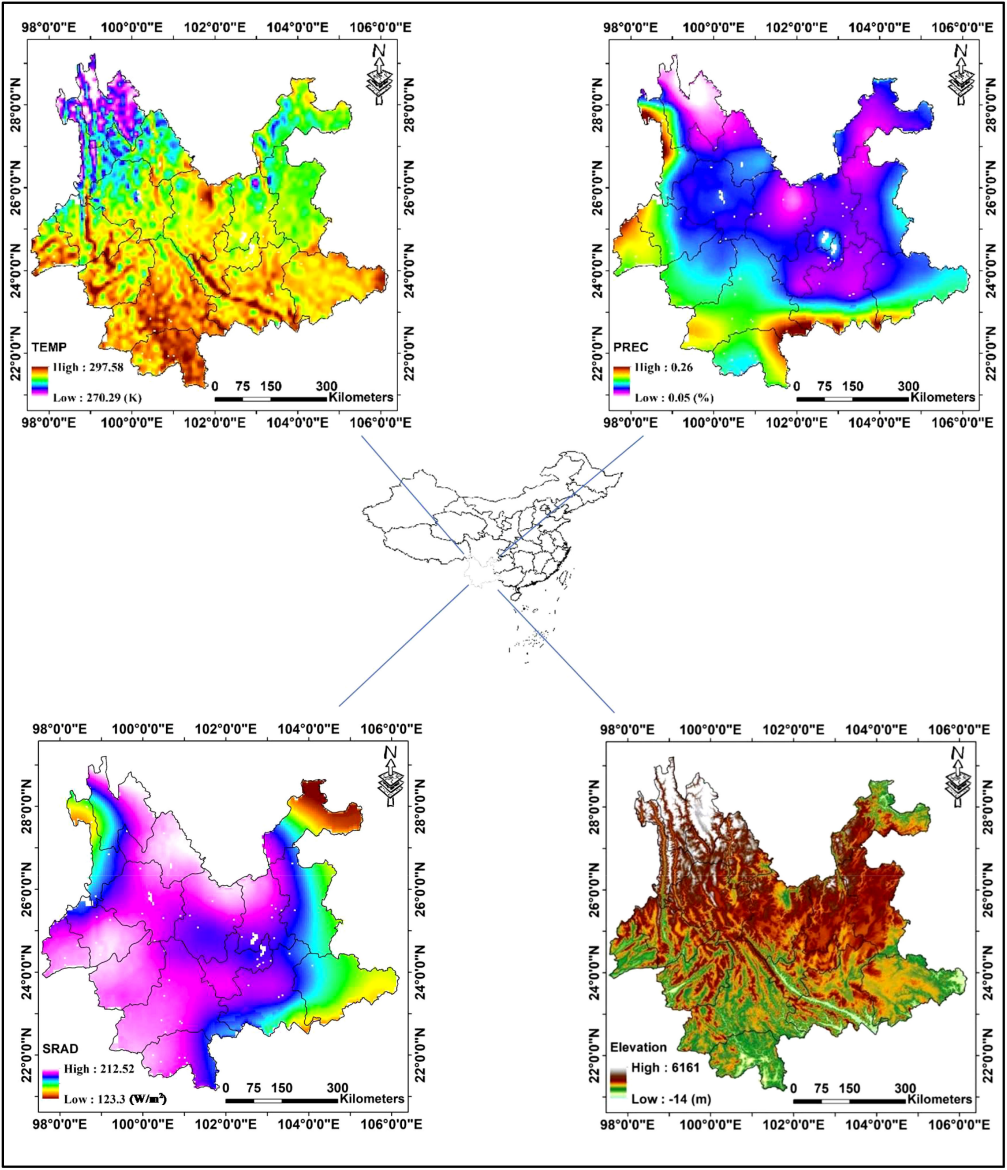
Location of the study area: inset maps show mean annual temperature (TEMP), mean annual precipitation (PREC), and mean annual solar radiation (SRAD) during 2001–2018, as well as digital elevation (Elevation).

Yunnan province (**Figure 1**), situated in the southwestern region of China, is renowned for its rich plant diversity and endemism. It encompasses a wide range of vegetation types, ranging from tropical rainforests to alpine shrublands. The distinct geographic location of Yunnan, where the Himalayan mountains and the Southeast Asian peninsula converge, makes it a critical area for studying the impacts of climate change on vegetation phenology. Firstly, Yunnan plays a vital role in the global carbon cycle, given its vast expanse of forests and other vegetation acting as carbon sinks (Yuan et al., 2017; Ding et al., 2021). Secondly, Yunnan exhibits a high sensitivity to variations in temperature and precipitation, which can profoundly impact vegetation phenology and ecosystem function (Ge et al., 2021). Thirdly, Yunnan province is renowned for its remarkable diversity in phenological patterns (Liu et al., 2022). The major vegetation types in Yunnan include tropical rainforest, monsoon rainforest, evergreen broad-leaved forest, coniferous forest, subalpine meadow, etc., each exhibiting distinct phenological characteristics. Changes in vegetation phenology and carbon uptake in Yunnan can therefore have significant implications for regional and global C cycling and climate. Overall, Yunnan’s rich plant diversity, sensitivity to climate variability, and ecological importance make it an ideal study object for advancing our understanding of the impacts of vegetation phenology on ecosystem function and service. Moreover, the unique characteristics of Yunnan can also provide insights into the broader global patterns of plant response and adaptation to climate change.

### 2.2 Data

Based on the research objectives of this paper and existing studies (Lu et al., 2021; Zhang et al., 2021; Chen and Zhang, 2023), we selected representative influencing factors of climate, phenology and vegetation growth conditions, respectively. The three meteorological variables were precipitation (PREC), temperature (TEMP) and solar radiation (SRAD). The three phenology variables were the start (SOS), end (EOS) and length (LOS) of the growing season. The normalized vegetation index (NDVI) was used to characterize the natural growth of vegetation. Due to the drastic changes in vertical elevation in Yunnan Province, this study specifically included elevation above sea level (ELEV) in the analysis. The researchers downloaded time series data (e.g., remote sensing and meteorological data) and non-time series data (e.g., elevation data) and pre-processed them, including coordinate alignment, projection transformation and resolution adjustment. Among them, the meteorological data (1979–2018, spatial resolution 0.1°) were obtained from the National Meteorological Data Center (https://data.cma.cn/) and the National Tibetan Plateau Data Center (https://data.tpdc.ac.cn/). MODIS satellite data (2001–2019, spatial resolution 500 m) MODIS satellite data (2001–2019, spatial resolution 500 m), including MOD09A1-(NDVI), MCD12Q2-(LOS/EOS/SOS) and MOD17A3-(NPP). Digital elevation data (90 m spatial resolution) were downloaded from the USGS (https://lpdaac.usgs.gov/data/). Among the data products, the Terra and Aqua combination Moderate Resolution Imaging Spectroradiometer (MODIS) Land Cover Dynamics (MCD12Q2) version 6 data product provides global land surface phenology metrics for each year from 2001 through 2019. The MCD12Q2 Version 6 data product is derived from time series of the 2-band Enhanced Vegetation Index (EVI2) calculated from MODIS Nadir Bidirectional Reflectance Distribution Function (BRDF)-Adjusted Reflectance (NBAR). Vegetation phenology metrics at 500 meter spatial resolution are identified for up to two detected growing cycles per year. Each MCD12Q2 file (multilayer data file HDF4 format) provides information layers of detected phenological indicators on an annual basis. For example, total number of vegetation cycles, green start (i.e., SOS), green rise midpoint, maturity, green peak, senescence, green fall midpoint, and dormancy (i.e., EOS). And LOS is obtained by subtracting SOS from EOS. Due to the limitations of MODIS data and meteorological data time series, the time span used in this project is 2001–2018. The temporal resolution of all time series data is in years. Additionally, given the large number of SEM models, the long computation time, and the spatial resolution of all the data, we used the “cubic” method to resample the data and increase/decrease the spatial resolution to 5000 m to improve computational efficiency.

### 2.3 Methods

#### 2.3.1 Pearson correlation

We used Pearson correlation to detect the correlation between the factors. The Pearson correlation formula can be seen in Eq. (1):


(1) $$r=\frac{n\sum^{}xy-\left(\sum^{}x\right)\left(\sum^{}y\right)}{\sqrt{\left[n\sum^{}x^{2}-{\left(\sum^{}x\right)}^{2}\right]\left[n\sum^{}y^{2}-{\left(\sum^{}y\right)}^{2}\right]}},$$

where *r* is the Pearson correlation coefficient, *x* represents the values in the first set of data, *y* represents the values in the second set of data, and *n* is the total number of values. The *r* is a number between −1 and +1 that measures the strength and direction of the relationship between two variables.

#### 2.3.2 SEM

We use Structural equation modeling (SEM) to decompose the direct and indirect effects of the factors. SEM combines traditional path analysis and factor analysis perfectly and enables the decomposition of direct and indirect effects of one variable on another, representing them as standardized path coefficients (Sun et al., 2022). In the model, the path coefficient represents the magnitude of the direct effect, whereas the product of all path coefficients along the pathway from the dependent variable to the outcome variable through mediating variables signifies the magnitude of the indirect effect. The total effect is obtained by summing the direct and indirect effects. The SEM model is defined as follows:


(2) y=By+Γx+ζ

where *y* and *x* indicate the column vectors of the endogenous variables and column vectors of the exogenous variables, respectively. B, Γ, and ζ are the relationships between the endogenous variables, the influence of exogenous variables on the endogenous variables, and the residual term of the structural equation, respectively. All statistical analyses were performed using the Amos 24 software (IBM SPSS Inc.). To analyze the direct effect of climate on NPP and the indirect effect of climate on NPP through phenology and vegetation growth status, we constructed four types of SEM models for the analysis (Chu et al., 2016). First, multicollinearity among the factors was tested before modeling, and the test results are given in **Table 1** in the supplementary document. Among them, LOS had significant multicollinearity problems with SOS and EOS. So LOS was modeled separately from EOS and SOS factors. Secondly, four types of SEMs were constructed: (1) SEM1 with only ELEV and climate factors, (2) SEM2 with the introduction of the vegetation growth condition factor NDVI in addition to SEM1, (3) SEM3 with the introduction of the vegetation phenology factor in addition to SEM1, and (4) SEM4 with both the vegetation growth condition factor NDVI and the phenology factor introduced in addition to SEM1.

**Table 1:** Results of multicollinearity detection (MLD) for each factor.

MLD	TEMP	PREC	SRAD	SOS	EOS	LOS	NDVI	ELEV
VIF of all factors without LOS	3.99	1.64	1.32	2.39	1.95	–	2.32	4.67
VIF of all factors without SOS and EOS	3.99	1.63	1.23	–	–	2.33	2.09	4.42
VIF of all factors	4.0	1.7	1.33	267.51	420.92	491.79	2.39	5.1

VIF<5, no significant multicollinearity problem.

#### 2.3.3 Polynomial regression model

We used a polynomial regression model to fit the changes in the factors. The use of linearity or non-linearity was determined by looking at the scatter plots of the factors, as well as the *R*-squared and *p*-values of the regression results. Such a model for a single predictor, *X*, is:


(3) $$\text{Y}={\text{β}}_{0}+{\text{β}}_{1}\text{X}+{\text{β}}_{2}{\text{X}}^{2}+\ldots +{\text{β}}_{h}{\text{X}}^{h}+\in ,$$

where *h* is called the degree of the polynomial. For lower degrees, the relationship has a specific name (i.e., *h* = 2 is called quadratic, *h* = 3 is called cubic, *h* = 4 is called quartic, and so on).

#### 2.3.4 Variance inflation factor

We used variance inflation factors (VIF) to detect multicollinearity among the factors. The VIF estimates how much the variance of a regression coefficient is inflated due to multicollinearity in the model. VIF for the *j^th^* predictor is:


(4) $$VIF_{j}=\frac{1}{1-R_{j}^{2}}$$

where $R_{j}^{2}$ is the *R^2^*-value obtained by regressing the *j^th^* predictor on the remaining predictors. A *VIF* of 1 means that there is no correlation among the *j^th^* predictor and the remaining predictor variables. The general rule of thumb is that *VIF_j_* exceeding 5 warrant further investigation, while *VIF_j_* exceeding 10 are signs of serious multicollinearity requiring correction (Marcoulides and Raykov, 2019).

#### 2.3.5 Slope (trend analysis)

We use slope to determine the trend of climate factors over the time series. The slope of the fitting function can be expressed as follows (Ge et al., 2021; Wu et al., 2021; Zhang and Ye, 2021):


(5) $$Slope=\frac{n\sum_{i=1}^{n}i\times X_{i}-\sum_{i=1}^{n}i\sum_{i=1}^{n}X_{i}}{n\sum_{i=1}^{n}i^{2}-{\left(\sum_{i=1}^{n}i\right)}^{2}},$$

where the *slope* is the trend of the factor in time series, *n* is the number of years monitored, and *X_i_* represents the element value corresponding to the *i*^th^ year. When the *slope*>0, the elemental sequence increased across the time steps; conversely, when the *slope*<0, the elemental sequence decreased, and a larger absolute value of the *slope* resulted in a greater rate of change of the element.

## 3 Results

### 3.1 Spatio-temporal variations in climate

**Figure 2:**
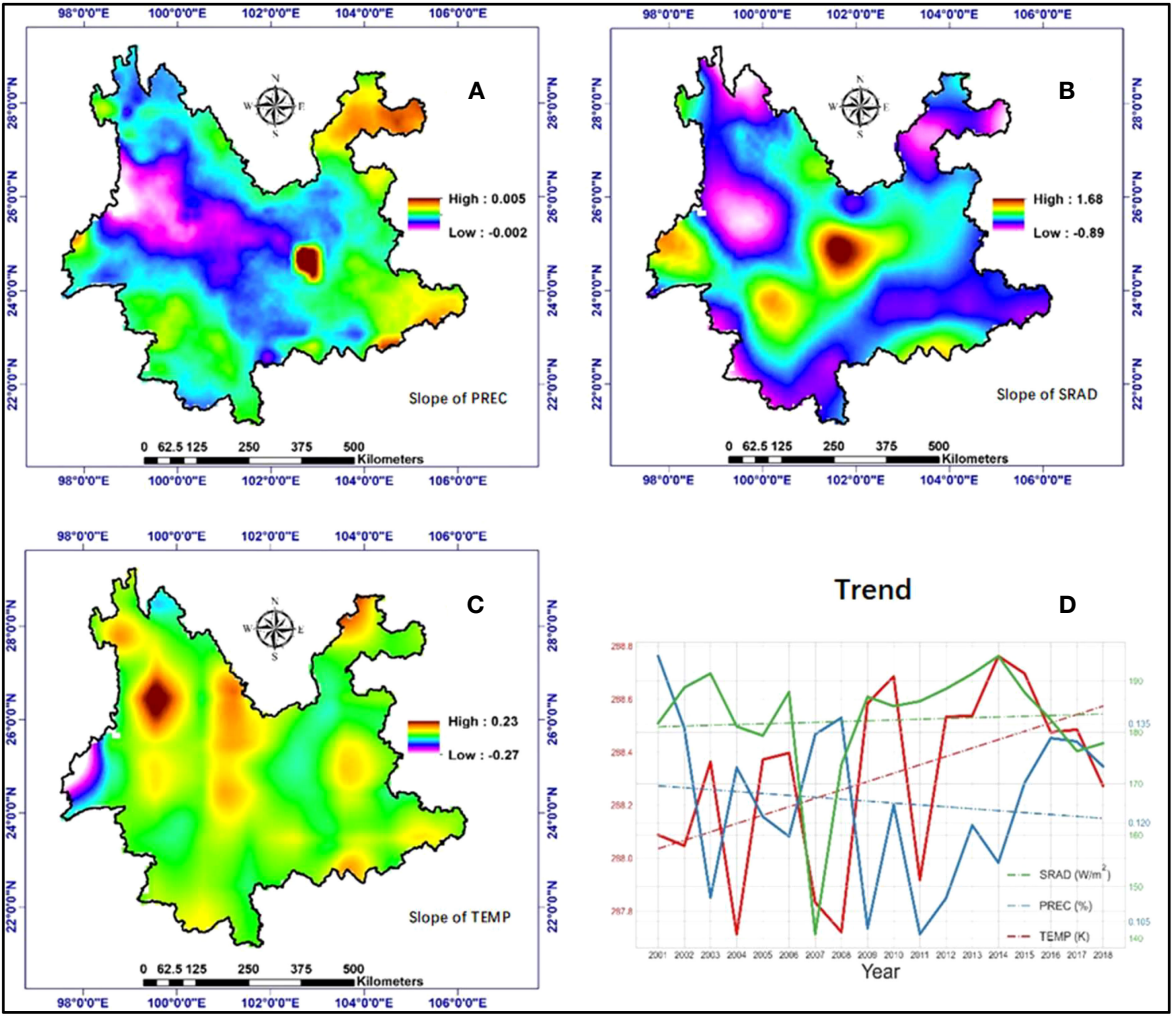
Climate change trends. Changes in **(A)** slope values of precipitation (PREC), **(B)** slope values of solar radiation (SRAD), **(C)** slope values of temperature (TEMP), and **(D)** trends of PREC, SRAD and TEMP during 2001–2018.

The overall temperature in Yunnan Province is high in the south and low in the north, consistent with the latitudinal distribution. Northwestern Yunnan has lower temperatures due to the effect of higher elevation. On the other hand, some low-lying areas, such as dams and river valleys (**Figure 1** Elevation), have higher temperatures (**Figure 1** TEMP). Rainfall in Yunnan Province is influenced by the monsoon climate zone in which it is located, with a general distribution of high in the southwest and low in the northeast. Also influenced by the monsoon, southeastern Yunnan has relatively high rainfall. Moreover, due to the influence of altitude, rainfall in the upland region is lower than that in the lowland region (**Figure 1** PREC). In particular, under the combined effects of location, monsoon, topography and sunshine hours, solar radiation in Yunnan Province shows a characteristic mid-high and two-side-low distribution form. At the same time, the area with higher radiation in the middle shows a distribution form of high in the north and south and low in the middle due to the topography (**Figure 1** SRAD). From **Figure 2**, it can be seen that the climate of Yunnan Province has a certain trend over the study period. Temporally, temperature showed a significant upward trend (slope = 0.032, P = 0.035), whereas neither rainfall nor solar radiation changes were significant (**Figure 2D**). Spatially (based on each pixel), the trend in rainfall exhibited a minor magnitude (slope ranging from −0.002 to 0.005) and indicated a slight decrease in the northwestern region (**Figure 2A**). Conversely, the trend of solar radiation displayed the largest magnitude (slope ranging from −0.89 to 1.68) and was more variable in the study area. Specifically, the outer regions of Yunnan Province were dominated by decreasing trends, while the central part was dominated by increasing trends (**Figure 2B**). Temperature trends were moderate and relatively consistent within the study area, except for a significant decreasing trend in the western margin and a significant increasing trend in the northwestern part (**Figure 2C**).

### 3.2 Correlation among phenology, climate, and NPP time series

**Figure 3:**
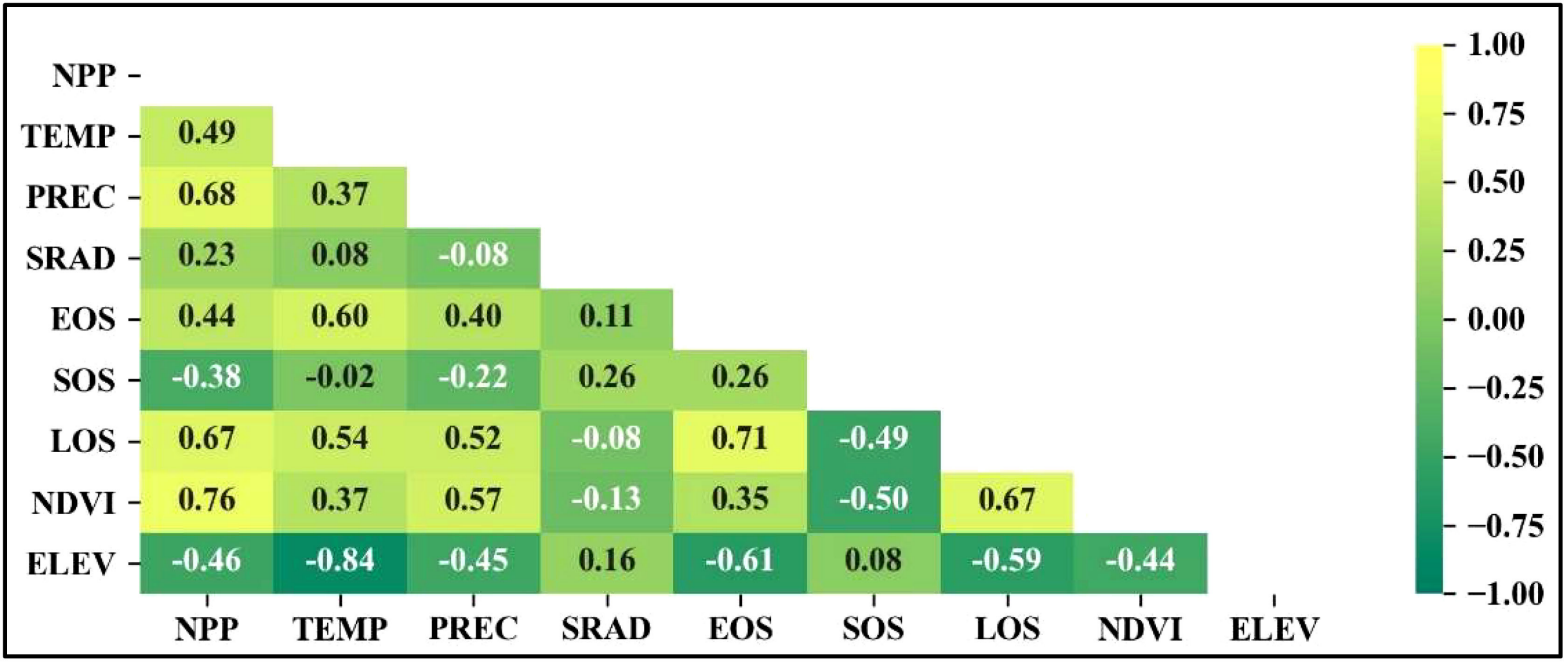
Correlations between climate and NPP and phenology during 2001–2018.

To analyze the relationship between the climate and NPP and phenology over time, we performed a correlation analysis for the 2001–2018 time series (**Figure 3**). The *p*-values for all correlations were less than 0.05. Among them, NPP was mainly positively correlated with PREC, LOS and NDVI, with correlation coefficients of 0.68, 0.67 and 0.76, respectively. LOS was mainly positively correlated with TEMP, PREC, EOS and NDVI, and negatively correlated with ELEV, with correlation coefficients of 0.54, 0.52, 0.71, 0.67 and −0.59, respectively. SOS was mainly negatively correlated with NDVI, with a correlation coefficient of −0.5. EOS was mainly positively correlated with TEMP, and negatively correlated with ELEV, with correlation coefficients of 0.6 and −0.61, respectively. The climate factors that have the greatest impact on NPP, EOS/LOS, SOS and NDVI are PREC, TEMP, SRAD and PREC, respectively.

### 3.3 Changes and relationships of SOS, EOS and LOS under global warming

**Figure 4:**
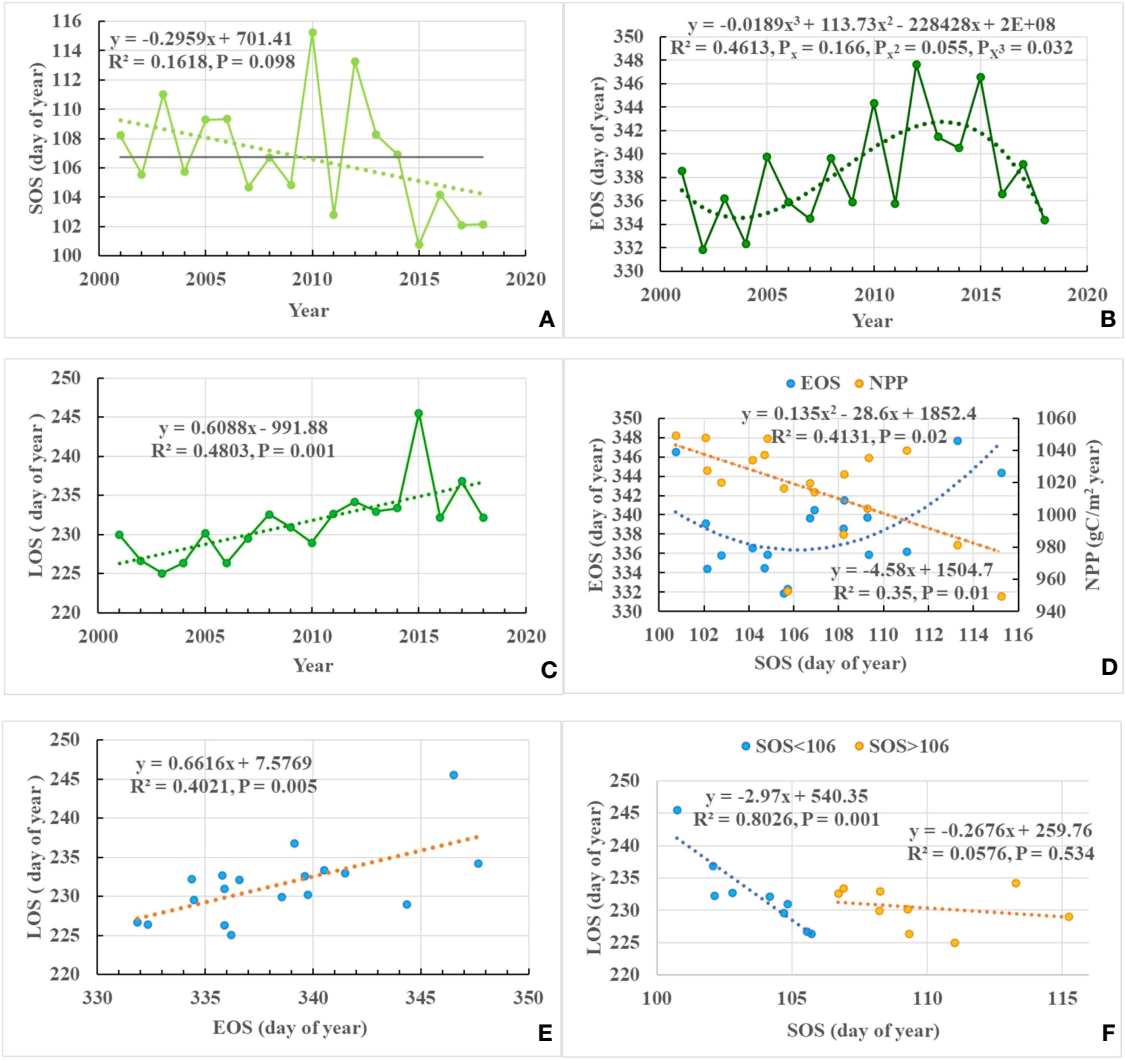
Relationship between the start (SOS) and end (EOS) of the growing season for 2001–2018. Temporal trends in: **(A)** the start of the growing season (SOS), **(B)** the end of the growing season (EOS), and **(C)** the length of the growing season (LOS). Relationship between: **(D)** net primary production (NPP), the end of the growing season (EOS) and the start of the growing season (SOS), **(E)** the end of the growing season (EOS) and the length of the growing season (LOS), and **(F)** the start of the growing season (SOS) and the length of the growing season (LOS).

SOS showed a certain trend of advancement over time, although the trend was not very significant and only satisfied a significance of 0.1 (**Figure 4A**). On the other hand, EOS did not show a significant linear relationship over time. However, it satisfied a significant cubic relationship, forming a fluctuating change of advancement, followed by postponement and then advancement again (**Figure 4B**). While both SOS and EOS did not show significant linear changes over time, LOS showed a significant linear growth trend (**Figure 4C**). The SOS and EOS show a clear quadratic U-shaped relationship (**Figure 4D**). As shown in the figure, when the SOS is less than 106 day of year, a delayed SOS will advance the EOS, resulting in a shorter LOS. Conversely, when the SOS is greater than 106 day of year, the EOS will also be delayed if the SOS is delayed. However, since the delay in the EOS is greater than the delay in the SOS, it leads to an increase in the LOS. Consequently, the non-linear relationship between SOS and EOS leads instead to a very significant linear growth trend in LOS. Furthermore, it is evident from the figure that the NPP exhibits a notable decrease with a delay in the SOS. Specifically, for each day of SOS delay, the NPP decreases by 4.58 gC/m^2^ yr. LOS is jointly determined by SOS and EOS. Specifically, LOS exhibits a highly significant positive correlation with EOS across years. For every day of delay in EOS, LOS will lengthen by 0.66 days/year (**Figure 4E**). Unlike EOS, SOS has a segmented effect on LOS. When SOS is less than 106 day of year, SOS has a significant impact on LOS. Every day of delay in SOS reduces the LOS by 2.97 days (**Figure 4F**). However, when SOS is greater than 106 day of year, the effect of SOS change on LOS is significantly attenuated and the change in LOS is mainly determined by EOS (**Figures 4E, F**).

### 3.4 Structural equation model results

**Figure 5:**
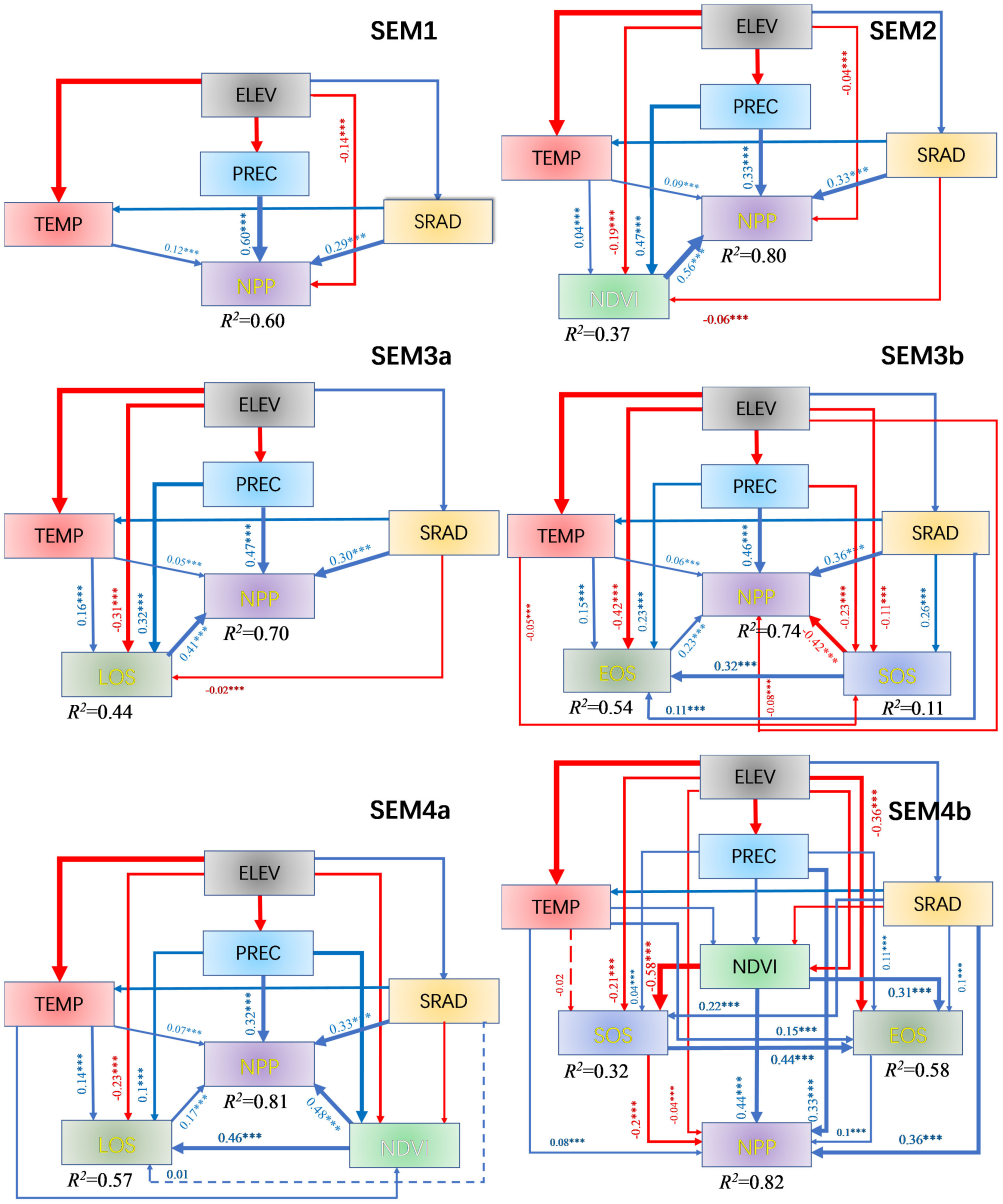
Structural equation modeling (SEM) is utilized to analyze the direct, indirect and total effects of the variables. χ^2 ^= 4.65, df = 2, p = 0.1, RMSEA<0.01; SEM1: AIC = 30.65; SEM2, SEM3a: AIC = 42.65; SEM3b, SEM4a: AIC = 56.65; and SEM4b: AIC = 72.65 (As new variables are introduced, the AIC values become progressively larger). The red and blue arrows indicate negative and positive correlations, respectively, and arrow thickness is the strength of correlation. The solid and dotted lines indicate significant (*p<*0.05) and insignificant effects (*p* > 0.05), respectively. Values on the arrows indicate standardized path coefficients. The *R*^2^ values below the response variables represent the proportion of variation explained by relationships with other variables. ***, *p* < 0.001.

As shown in **Figure 5**, the explanatory power of ELEV and climate factors for NPP is 0.6 (SEM1). The explanatory power for NPP reaches 0.8 (SEM2) when NDVI is added to the model SEM1. However, introducing SOS-EOS or LOS as phenological factors only yields an explanatory power for NPP ranging from 0.7 to 0.74 in SEM3. When both NDVI and phenological factors SOS-EOS or LOS are included in the model (SEM4), the explanatory power for NPP demonstrates a minimal increase of 0.01–0.02 to reach 0.81–0.82 compared to that in SEM2. In addition, the direct effects of PREC and TEMP on NPP were significantly reduced by adding NDVI and phenological factors and then decomposed into indirect effects, while the direct effects of SRAD on NPP were increased. For instance, (1) When we introduced the EOS-SOS or LOS factor (SEM3), the indirect influence of PREC on NPP through phenology was 0.134 (EOS-SOS) and 0.132 (LOS), splitting out 22.4% and 22.1% of the total effect, respectively. The indirect effects of TEMP were 0.053 and 0.065, splitting 45.3% and 55.6% of the total effect, respectively. (2) When we introduced the NDVI factor (SEM2), the indirect effect of PREC was 0.262, accounting for 43.9% of the total effect, while the indirect effect of TEMP was 0.024, splitting 20.5% of the total effect. (3) When both phenology (EOS-SOS/LOS) and NDVI factors were introduced (SEM4), it combined the partitioning effect of both types of factors on PREC and TEMP, although the total effect on NPP did not increase. That is, the indirect effect of PREC on NPP was 0.267 (EOS-SOS&NDVI) and 0.279 (LOS&NDVI), splitting 44.7% and 46.7% of the total effect, respectively. The indirect effects of TEMP on NPP were 0.041 and 0.047, splitting 35% and 40.2% of the total effect, respectively. (4) The direct effect of SRAD increases from 0.29 in SEM1 to 0.36 in SEM4b. It is worth noting that although the SOS-EOS model exhibits slightly higher ultimate explanatory power for NPP than the LOS model, the latter demonstrates a lower Akaike Information Criterion (AIC) value.

## 4 Discussion

### 4.1 Changes in phenology and the influence of climate on phenology

**Figure 6:**
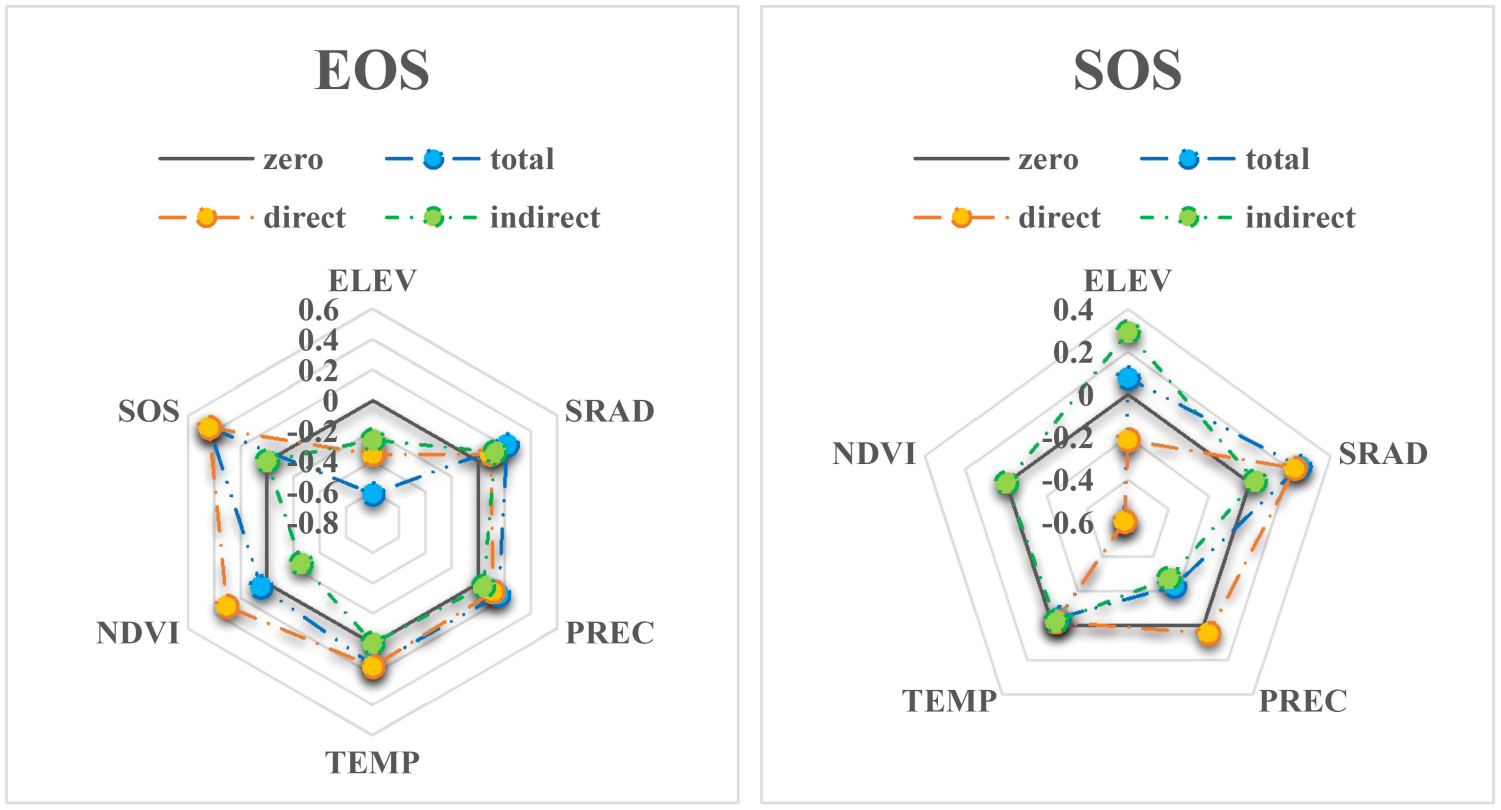
Direct, indirect and total effects of each factor on EOS and SOS in SEM4.

Among the climate factors, TEMP mainly affects EOS directly (**Figure 6** EOS), SRAD mainly affects SOS directly, and PREC mainly affects SOS indirectly (**Figure 6** SOS), while PREC and TEMP have no significant direct effect on SOS. Similarly, Chen and Zhang (2023) found that in Yunnan, the temperature was significantly positively correlated with EOS and SRAD was significantly positively correlated with SOS. Zhang et al. (2023) also found that temperature had a greater effect on autumn phenology than radiation and precipitation in China. In contrast to our study, Wu et al. (2021) found that SOS was significantly negatively correlated with temperature in most of the study area, and Shen et al. (2022) also found that SOS in the study area was more sensitive to mean temperature and warming in both time and space, while Yu et al. (2022) found that the main trends of SOS and EOS in the study area were closely related to changes in temperature and precipitation.

Furthermore, we found that the overall effect of SRAD on SOS and EOS was the largest among the climate factors (**Figure 6**). Stronger SRAD is usually accompanied by higher temperature and sufficient sunshine intensity (Yang et al., 2017), so in addition to its role in photosynthesis, it can provide valuable insights into photoperiod estimation (Yang et al., 2017) and indicate the duration of sunlight at sunrise. Stronger SRAD is associated with higher sunshine intensity, higher temperature and longer sunshine duration (Calle et al., 2010). This suggests that SRAD is the most important climatic factor determining SOS and EOS in Yunnan Province. These results are consistent with those of Chen and Zhang (2023). In addition, although TEMP may not have as much influence on SOS as PREC and SRAD, it still plays an important role. Specifically, TEMP determines the onset of photoperiodic signal capture. Since autumn and spring have the same photoperiod length, plants must first experience low temperatures and then receive signals that winter has passed before they can begin to receive photoperiodic signals (Körner and Basler, 2010). Photoperiod is defined as the duration of light availability in a 24-hour period (Piao et al., 2019), and changes in photoperiod are relatively more accurate and stable than changes in temperature. Yunnan Province has a complex and diverse topography, and temperature varies dramatically. If the vegetation SOS is only sensitive to temperature, it is easy to make “mistakes”. Photoperiod sensitivity can, therefore, protect plants from the potentially fatal consequences of tracking temperatures at the “wrong” time of year (Körner and Basler, 2010). As a result of global warming, plants may sense temperature increases earlier and thus emit SOS earlier; however, future prolonged and rapid warming may increase the thermal requirements for SOS by reducing the number of cold days or slowing the realization of cold requirements, potentially slowing the trend of SOS progression (Shen et al., 2022). As the SOS photoperiod threshold (set by genes) is approached, internal controls will increasingly limit the progressive trend of SOS. When the photoperiod threshold is reached, the SOS will stop advancing, regardless of increasing temperature or solar radiation, due to internal genetic limitations. Breaking this limit will require several generations of trees to renew the mutation, which could take hundreds of years. This phenomenon may provide some insight into the slowing of phenological change in most vegetation (Körner and Basler, 2010).

In addition, the negative correlation between PREC and SOS may also be caused by increased precipitation leading to increased cloudiness, which in turn reduces SRAD and indirectly affects SOS. It is also worth noting that the photoperiodic sensitivity of spring phenological events may vary considerably between species and latitudes, and the influence of climate on phenology may also vary considerably between regions (Piao et al., 2019). As the exact mechanisms of the effects of temperature, precipitation and their interactions on phenology are incompletely understood (Yu et al., 2022), and the responses of different ecosystems to different levels of climate change are far from clear, the main reasons for these differences need to be further investigated and analyzed, especially in the Chinese region (Yang et al., 2017).

### 4.2 Interrelationships and effects of vegetation phenology factors

The spatial pattern of EOS trends is more complicate than that of SOS (Piao et al., 2019). EOS is positively correlated with TEMP and shows significant cubic polynomial fluctuations over time due to the very drastic temperature changes in Yunnan Province. Consistent with our findings, Shen et al. (2022) also observed that higher *T_min_* (Minimum Temperature) substantially delays the EOS by slowing the physiological processes of leaf senescence. However, Wu et al. (2021) found that the autumn phenology of typical vegetation types in the study area was advanced significantly, even more than the spring phenology. In addition, recent studies found a positive intercorrelation between spring and autumn phenology (Piao et al., 2019; Chen and Zhang, 2023). Nonetheless, our study found a significant quadratic U-shaped relationship between SOS and EOS (**Figure 4D**). When SOS is greater than 106 days, the direction of change of SOS and EOS is primarily synchronous, i.e. if SOS is postponed, EOS is also delayed. Combined with **Figures 4D–F**, it becomes evident that once SOS is above 106 days, there is no longer a significant relationship between SOS and LOS, and the prolongation of LOS is mainly dominated by EOS. Therefore, with the aging of the plant leaves and increased respiration due to delayed EOS, NPP decreased instead. However, when SOS was less than 106 days, the changes in SOS and EOS became opposite, i.e. SOS advanced, EOS delayed, and LOS prolonged, resulting in an increase in NPP. Shen et al. (2022) also showed that EOS was affected by SOS, which was manifested in the form of EOS advancing (delayed) as SOS advanced (delayed). Warmer temperatures and increased precipitation were shown to be the main drivers of SOS advancement and EOS delay, but insufficient moisture led to SOS delay and EOS advancement. Shen et al. (2022) found that although the direct effect of temperature on plant phenology is stronger than that of moisture conditions, the deterioration of moisture conditions due to warming may weaken (or even reverse) the effect of temperature on phenology. Coincidentally, the mean value of SOS over the study period was also about 106 days. This means that the mean value of SOS (106 days) is likely to be the dividing line between changes in NPP (increase or decrease) due to changes in EOS (advance or shift) in the later period, which represents a transition point in the biological growth and maintenance process in Yunnan Province. In addition, the SOS in Yunnan Province maintained a continuous advancement from 2012 to 2015, and the changes in SOS and EOS became opposite after 2014 (**Figures 4A, B**). This change may be related to the consecutive province-wide drought disasters in Yunnan from 2009 to 2013. Annual precipitation was more than 5% below normal in each of these five years, with three consecutive years of 14% below normal in 2010, 2011 and 2012. There is some evidence that warmer or earlier springs may cause earlier autumn senescence because of the fixed lifespans of leaves or adversely affect plant productivity later in the season through the build-up of water deficits (Buermann et al., 2018; Liu et al., 2022). Shen et al. (2022) also found that the EOS tends to advance under higher *T_max_* (Maximum Temperature), likely owing to confounding water limitation. Because earlier spring phenology may increase soil water loss in the early stages of the growing season, thereby increasing the prevalence of summer drought that may subsequently result in earlier leaf senescence (Piao et al., 2019). Li et al. (2023) also found that Spring phenology strongly governs vegetation growth and soil water consumption in the growing season. Vegetation phenology interacts with drought timing and thus determines subsequent vegetation growth. Furthermore, the interaction between spring and autumn phenological events is likely to modify phenological responses to ongoing climate warming (Piao et al., 2019). When the water deficit due to the advancement of SOS reaches the threshold of vegetation water or nutrient tolerance, EOS no longer postpones but starts to advance with the advancement of SOS (Shen et al., 2022). Possible underlying mechanisms for such interseasonal phenological correlations may be directly related to leaf traits and may be ascribed to the indirect effects of environmental factors as well (Piao et al., 2019). Because the SOS is involved in plants’ life rhythm and carbon storage, it could modify the matching between environmental conditions and life-cycle stages (Shen et al., 2022). However, the relative importance between biological factors and environmental factors on phenological responses to climate warming needs to be further investigated (Piao et al., 2019). Likewise, Xiong et al. (2023) found that spring phenology trends in northern hemisphere mid- and high-latitude vegetation showed an “early to late” reversal during the warming stagnation period, and the average rate of change around the turning point was small. The non-linear nature of this change invalidates the linear trend analysis often used in existing studies, leading to important differences in the analysis of spring phenology trends in the northern hemisphere mid- and high-latitude vegetation during the warming stagnation (Xiong et al., 2023). The timing of plant phenology events is determined by a variety of biological and environmental factors. However, the extent to which these factors influence plant phenology depends largely on the different developmental stages of phenological events and specific differences in plant life history strategies. Therefore, the relative importance of biological and environmental factors on the phenological response to climate warming requires further research in the future (Piao et al., 2019).

As seen in **Figure 4**, while neither SOS nor EOS showed a significant linear change, LOS maintained a significant linear growth trend during the study period. On the one hand, although there is advancement in EOS, the advancement trend of SOS is stronger and there is an enhanced influence of SOS on LOS. Piao et al. (2019) also found that the increase in LOS is mainly driven by the advancement of SOS in Eurasia. On the other hand, because SOS has not yet reached the photoperiodic threshold, there is still room for SOS advancement. It can be seen that LOS in Yunnan is mainly dominated by both SOS and EOS in the early stage and by SOS in the later stage. Additionally, although NDVI and TEMP are the main influences on LOS, PREC has a significant nonlinear effect.

### 4.3 Impact of phenology on the relationship between climate and NPP

**Figure 7:**
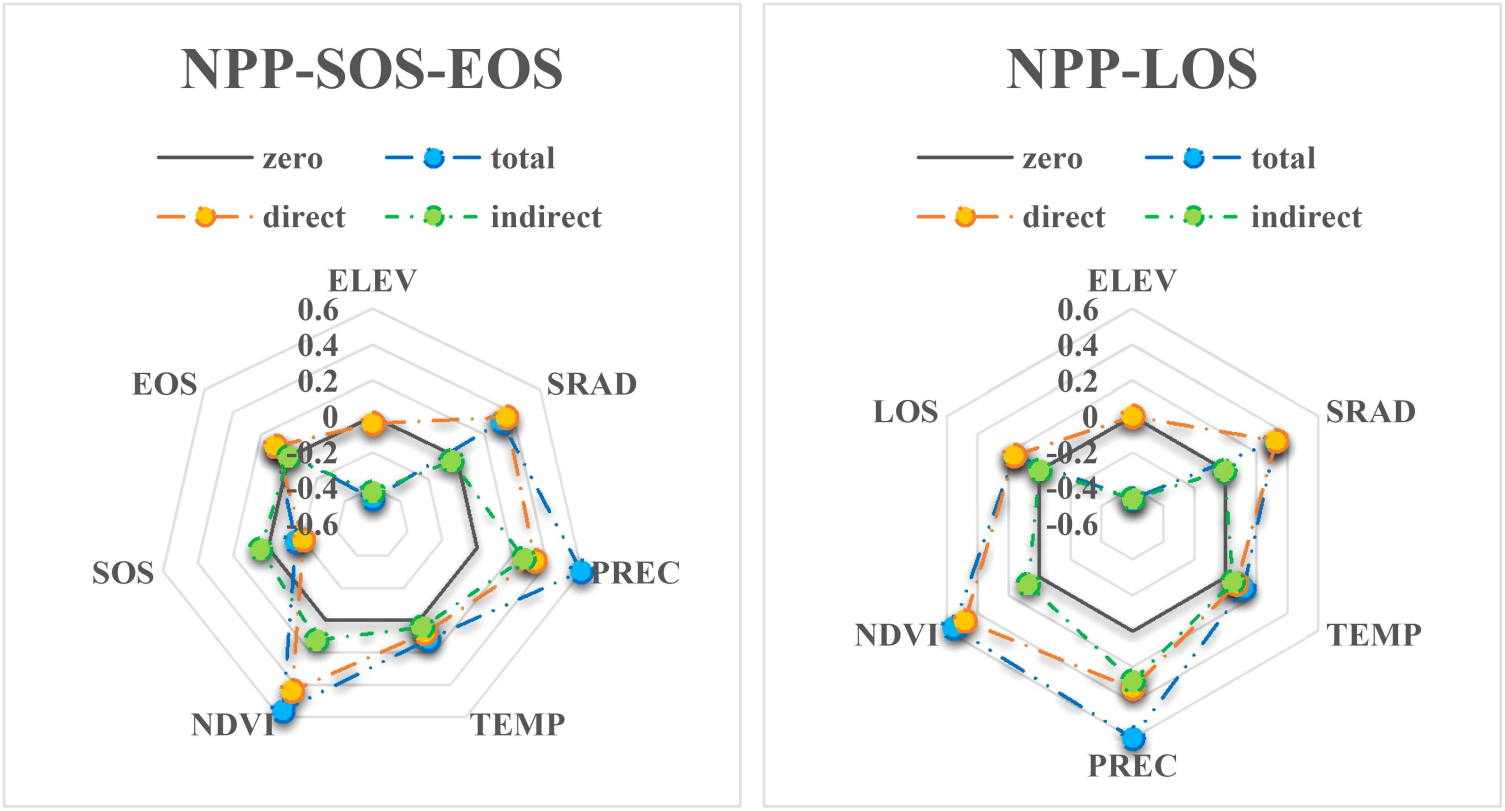
Direct, indirect and total effects of each factor on NPP in SEM4.

**Figure 7** shows that NDVI, SRAD, SOS, EOS, and LOS have mainly direct effects on NPP. ELEV has mainly indirect effects on NPP. PREC and TEMP have a mixture of direct and indirect effects on NPP, and the indirect effect occurs mainly due to the introduction of NDVI or/and phenological factors. As reported by Chen and Zhang (2023), the interaction of PREC and TEMP with other factors had a large effect on NPP. Moreover, in our study the effect of LOS on NPP was slightly lower than that of SOS-EOS on NPP, which differs from the results of most studies. LOS is jointly constrained by both SOS and EOS, and although LOS showed a significant and continuous increasing trend, the changes in SOS and EOS were more complex, as mentioned above (**Figure 4**). Therefore, SOS-EOS has a better explanatory power for the complex changes in NPP. Similar to our results, Yu et al. (2022) also found that SOS-EOS was more important than LOS. Although the delayed EOS led to a longer LOS, there was no increase in NPP due to the limited and reduced photosynthetic capacity coupled with an increase in respiration within the forest. Furthermore, besides SRAD being critical for phenology, Yang et al. (2017) found that the direct effect of solar radiation on NPP (0.31) was greater than precipitation (0.25) and temperature (0.07). Consistently, our findings also showed that the direct effect of SRAD (0.36) on NPP was greater than PREC (0.33) and TEMP (0.08). Notably, in the correlation analysis, the correlation between TEMP and NPP was much greater than that of SRAD. However, in the analysis of SEM, the direct effect of SRAD on NPP was much greater than that of TEMP. These results suggest that TEMP may not be the main direct limiting factor for vegetation NPP in Yunnan Province. Instead, the study found that SRAD may have a greater direct influence on NPP due to its contribution to photosynthesis. Similarly, the study of Chen and Zhang (2023) found that SRAD is one of the factors that has an important effect on NPP and should not be ignored. In addition, the relatively low effect of TEMP on NPP suggests that the direct effect of warming on NPP is relatively low. Nevertheless, other climate changes due to warming, like shifts in the timing, location, and intensity of extreme weather and precipitation, may considerably impact NPP (He et al., 2022).

Based on the SEM comparison (**Figure 5**), it was observed that the overall explanatory power of each climate factor on NPP remained unchanged (with PREC, TEMP, and SRAD exhibiting total influences on NPP of 0.597, 0.117, and 0.321, respectively). However, the introduction of phenology or NDVI altered the direct influence or explanatory power of the climate factors on NPP and resulted in the explanatory power of ELEV on NPP being almost completely dominated by indirect effects (**Figure 7**). Specifically, the direct influence of TEMP and PREC on NPP is weakened, but still greater than their indirect influence on NPP. On the other hand, the direct influence of SRAD is strengthened and becomes the dominant factor among the climate factors with direct influence on NPP. This result is mainly due to the fact that both TEMP and PREC influence NPP indirectly by affecting NDVI and phenology (Michaletz et al., 2014; Chu et al., 2016; Buermann et al., 2018; Piao et al., 2019). Among them, PREC affects NPP mainly by influencing the vegetation growth condition (NDVI) after SOS, because PREC has the largest influence on NDVI among the climatic and vegetation growth conditions (**Figure 3**). TEMP affects NPP mainly by changing LOS through EOS, because TEMP has the largest effect on EOS among the climatic and vegetation growth conditions (**Figure 3**). While SRAD directly affects SOS, the subsequent plant growth is mainly limited and affected by water, and the indirect effects of SRAD on NPP through phenology and NDVI have positive and negative effects that offset each other, so that the direct effects of SRAD on NPP are almost unaffected by the perturbation of phenology and NDVI. At the same time, because SRAD mainly affects SOS, and the effect of SOS on NPP is also largely dependent on subsequent PREC and NDVI, the indirect effect of SRAD on NPP is not significant. As SRAD is somewhat correlated with both TEMP and PREC (**Figure 3**), the weaker indirect effect of SRAD may have been included in the indirect effects of PREC and TEMP. Ultimately, we conclude that whether climate has an indirect effect on NPP is largely determined by the factors we have analyzed and the characteristics of the study area.

## 5 Conclusions

The mechanisms underlying the relationship between NPP and phenology are not fully understood, and several issues still need to be addressed. We utilized SEM modeling to examine how vegetation phenology affects the relationship between climate and vegetation NPP. Our analysis revealed the following conclusions: (1) TEMP mainly affects EOS directly, SRAD mainly affects SOS directly, and PREC mainly affects SOS indirectly. In addition, SRAD has the largest total effect on both SOS and EOS, indicating that SRAD is crucial to the phenology of vegetation in Yunnan Province. (2) There was a significant U-shaped relationship between SOS and EOS, and the change of SOS and EOS became opposite once SOS fell below 106 day of year. In addition, LOS maintained a significant linear increasing trend during the study period, while neither SOS nor EOS showed a significant linear change. (3) The direct effect of SRAD on NPP was greater than PREC and TEMP, indicating that SRAD may have a greater influence on NPP due to its contribution to photosynthesis. (4) PREC affects NPP mainly by influencing the vegetation growth condition (NDVI) after spring phenology (SOS); TEMP affects NPP mainly by influencing EOS to change LOS; while SRAD affects SOS directly in a photoperiodic manner and has a direct effect on NPP with little interference from phenology and NDVI.

However, our study has limitations due to the challenges and constraints in computation, data collection, and analysis methods. These limitations include: (1) the spatial and temporal resolution of the data used in our study are not high enough, which may lead to biased analysis results, especially at highly heterogeneous surfaces (Piao et al., 2019). (2) The time range of the analysis is not long enough, and an inadequate time range may not accurately reflect the actual patterns. Future research is necessary for continuous and long-term monitoring and analysis using satellite observations (Piao et al., 2019). (3) The resampling method may introduce uncertainty to the results (Shen et al., 2023).

## Data availability statement

Publicly available datasets were analyzed in this study. This data can be found here: https://lpdaac.usgs.gov/data/, https://data.tpdc.ac.cn/, https://data.cma.cn/.

## Author contributions

Conceptualization, YZ and XC; Methodology, YZ and XC; Software, XC; Validation, YZ; Formal Analysis, YZ and XC; Resources, YZ; Writing—Original Draft Preparation, XC; Writing—Review and Editing, XC and YZ; Funding Acquisition, XC and YZ. All authors contributed to the article and approved the submitted version.
